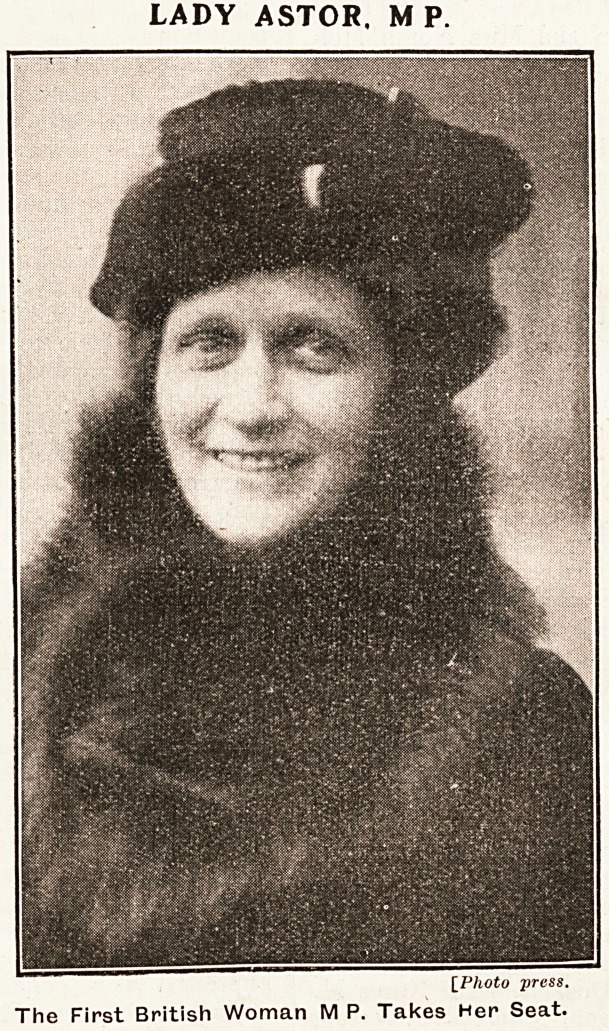# Round the Hospitals

**Published:** 1919-12-06

**Authors:** 


### ROUND THE HOSPITALS.

A development of the first importance was announced by Viscount Knutsford at the quarterly court of the Governors of the London Hospital on the 3rd instant. For many years the London Hospital has stood out for the contention that two years' training at the London Hospital is sufficient to make a competent trained nurse. Lord Knutsford has maintained this position for many years, and in his speech, which appears in another column, it will be seen he is at pains to make clear that in giving up his own personal feelings, and accepting the urgent wishes of the matron and Committee of the London Hospital to change the system of training from the two years of the past to the three years for the training of probationer nurses, which prevails throughout the principal British schools and training centres,

**ROUND THE HOSPITALS**—*(continued)*.

he has done so because they are about to try and shorten the hours of work of nurses at the London. To enable them to do this successfully they must evolve a plan which will necessitate lengthening the time of training. Lord Knutsford maintains that at the London Hospital they could train in two years while the nurses work the present number of hours, but when the hours are lessened they cannot get the training into two. The shortening of the hours compels Lord Knutsford to agree to change the system at the London to that of three years' training, and nothing except the force of circumstances would have induced him to adopt the change. He feels sure, however, and we incline to agree with him, that if the late Miss Luckes were alive to-day she would see the impossibility of shortening nurses' hours and training in the same period.

For our own part we congratulate the London Hospital on the change announced on Wednesday, and on the fact that under the new conditions which have arisen it has sunk its own opinion for the public good. This we know to be the policy which some of the best friends of the London Hospital have ever had at heart, and it augurs well for the future that this great hospital under its new matron should at length join hands with the majority of the nurse-training schools, and settle down to devote all its energies to the congenial task of making the training of nurses in this country the best in the world. Further, the aim will be that the conditions under which nurses are engaged, trained, employed, paid, housed and relaxed will be such as shall bring to these busy and devoted workers a continuous happiness in their work, the most vigorous and splendid health, whilst it keeps alight the spirit of devotion which they inherit from their great exemplar, the late Florence Nightingale. All interested in the London Hospital, and all who wish for the speedy and full development of nurse-training in this country, will recognise the courage and good judgment which Viscount Knutsford, the Chairman of the London Hospital, has shown in burying the hatchet and putting his shoulder to the wheel of progress for the benefit of all nurses wherever they are employed.

Mr. J. P. Lucas, Colonial Produce Broker, of Dunster House, Mincing Lane, E.C., has written a long letter to Dr. Addison, Minister of Health, " to mark the urgent need for legislature to enforce reforms in the conditions of the employment of probationers and nurses in hospitals, and to press the point that any measure of registration should extend to the institution of some authority to control the conditions under which probationers and nurses are to be employed in hospitals, with a view to the speedy abolition of the long prevailing system of excessive working hours or ' sweating.' " In the course of his letter Mr. Lucas falls into several errors, but his aim seems to be of the best, and he has elicited the following important reply from Dr. Addison:

" As you will doubtless have seen from Dr. Addison's speech on the second reading of the Bill he is fully alive to the necessity of improving the pay and conditions of employment of nurses; but the question is too complicated to admit of solution in the manner suggested by you, and the desired improvement can only be effected by stages. In any case the amendments for which you ask would not be in order on a Bill specifically limited to the registration of nurses, and they would involve separate legislation of a highly controversial character." All familiar with the actual position of matters affecting nurses at the present time will accept Dr. Addison's declaration as an encouragement to push forward with the object of securing, as speedily as possible, an improvement in the pay and conditions of employment of all trained nurses. This progressive spirit of justice and increase has been moving by leaps and bounds during the latter part of 1919. Indeed, so good is the progress and so sincere are the advocates and workers for expedition in this matter, that we are hopeful that a subject which in Parliament would almost necessarily prove of a " highly controversial character " may be happily and speedily overcome by private efforts and the co-operation of all the authorities immediately concerned.

The Nurses' Registration (No. 2) Bill passed through Standing Committee on Tuesday, December 2, and now awaits report to the House of Commons. Mr. T. P. O Connor, M.P. (the Father of the House), presided over the Committee, and amendments were down in the name of Sir Watson Cheyne, Dr. Addison, Mr. Johnstone, Mr. Kiley, Mr. Maddocks, Captain Elliot, Colonel Burn, and Mr. Robert Richardson, the first-named Member being responsible for the majority. An important amendment to the Bill was that made by Dr. Addison, that " where any person satisfies the conditions of admission to any supplementary or prescribed part of the Register, his or her name may be included in that part of the Register, notwithstanding that it is also included in the general part." Sir Watson Cheyne's amendment that a yearly fee of 2s. 6d. for the upkeep of the Register should not be imposed, but that a fine of 7s. 6d. should be substituted for a registered nurse's failure to notify the Registrar each year of her address, was withdrawn, Dr. Addison stating the yearly payment would do much to maintain a live Register.

In the debate on this amendment Dr. Addison made the interesting statement that he intended, in connection with grants to be made to institutions by the Ministry of Health, to enforce that such institutions must pay their nursing staff an adequate salary. This question is dealt with in its many aspects in our Foreword this week. An amendment by Captain Elliot to provide that, in the event of the establishment of a similar Council in Scotland or Ireland, the Councils shall confer in order to prescribe as far as possible identical rules throughout the three Kingdoms, was carried.

**ROUND THE HOSPITALS**—(*continued*).

Colonel Burn's amendment was withdrawn. It sought to secure representation on the Council of certain named Associations of children's hospitals, and that two of the sixteen nurses to be elected to the first elected Council should be trained in the nursing of sick children. Dr. Addison gave his assurance that the latter provision should be made, but stated that he could not run the risk of descending the slippery slopes of classification by mentioning the names of any other societies than those already included. On Sir Watson Cheyne's motion the title of the Bill was altered to read "A Bill to provide for the Registration of Nurses for the Sick."

THERE are a few people who seem to aim at circulating fairy-tales about the best of our hospitals without palpable reason or justification. St. Thomas's Hospital is the latest choice of the propagandist, who exhibits so small a measure of truth as to be laughable indeed. The following facts, which we owe to the courtesy of Miss Lloyd Still, Matron of St. Thomas's Hospital, cannot fail to interest our readers. At St. Thomas's Hospital no nurse has off duty for less than three and a-half hours daily, exclusive of meals, and in most departments it is for far longer. Twice in the week the senior nurse may remain on duty until 10 P.M. to give the sister's report to night nurses. It is one of the sternest and oldest rules that no meals are taken in the wards of the hospital. The ward kitchens are only serving rooms, of the same size as the sisters' room. The day nurses have their tea in this serving room for their own convenience, and the night nurses have their night meal and later refreshments there in absolute peace and quiet. Under no circumstances are they disturbed. In the special departments the nurses are not on duty at all until 3 P.M. on alternate Sundays.

NIGHT duty in St. Thomas's Hospital is proverbially light, there being no ward work (*i.e.* lavatories or bathrooms) undertaken, and only a small proportion of beds. Two nights off at the end of two months have been considered a better break than one night a month, but this point is under reconsideration. At the end of their three months' night duty each nurse has one night and one whole day off before returning to day work. All junior nurses are off at 8 A.M.; the senior nurse may remain until 8.15. Dinner is at 9 A.M., in mufti, if preferred, if a nurse wishes to go out immediately after. Many other readjustments are in being; the sisters' sitting-room is available for the use of the night nurses at night should they wish. Four years ago the nursing staff was doubled in every ward, resulting in considerable additional facilities for off-duty time. The holiday system at St. Thomas's is one of five weeks in the first year, *i.e.*, one week at the end of six months, and one month at the end of a year. In subsequent years it is four weeks, one in the spring and three in the summer. There is an alertness, brightness, and go about St. Thomas's Hospital sisters and nurses which impress a visitor and make him rejoice at the privilege, when duty brings it, of paying a visit to the wards and active working departments of this great Cure House on the Albert Embankment.

**LADY ASTOR, M P.**

[*Photo press.*

The First British Woman M P. Takes Her Seat.

THE King held two Investitures at Buckingham Palace last week. At the first, on November 26, His Majesty personally conferred decorations on members of the nursing services as follows:—Bar to the Royal Red Cross, First Class.—Sister Mildred Hughes, Q.A.R.N.N.S.; Royal Red Cross, First Class—Sister Mary Clark, Q.A.R.N.N.S.; Miss Betty Walker, Q.A.I.M.N.S.(R.), Miss Dorothy Foster, who also received the Military Medal, A.F.N.S.; Royal Red Cross, Second Class—Miss Mabel Bere, Q.A.R.N.N.S.; Miss Charlotte Robinson, who also received the Military Medal, Q.A.I.M.N.S.; Miss Edith Austin and Mrs. Florence Owen, Q.A.I.M.N.S.(R.), Miss Adelaide Bottrill, Miss Mary Edwards, Miss Beatrice Evans, Miss Mary Francis, Miss Elizabeth Martin, Miss Edith Pastfield, Miss Edith Porter, Miss Agnes Scott-Pullar, and Miss Elizabeth Woodward,

**ROUND THE HOSPITALS**—(*continued*).

T.F.N.S.; Miss Florence Corrigan. Miss Ina Dogherty, and Mrs. Elizabeth Panton, C.N.S.; Miss Nellie Coulson, B.R.C.S. His Majesty also conferred the Military Medal on Miss Louisa Gilbert, Q.A.I.M.N.S.(R.), and on Miss Katharine Freshfield, V.A.D.

At the second Investiture on November 27, the following members of the nursing services had the honour of receiving their decorations from the King:—Royal Red Cross and Bar—Mrs. Eva Pullinger, C.N.S.; Royal Red Cross, First Class—Miss Rose Lumsden, Q.A.I.M.N.S.(R.); Miss Enid Newton, T.F.N.S. Royal Red Cross, Second Class—Miss Winifred Halloran and Miss Norah Molloy, Q.A.I.M.N.S.; Miss Andrina Anderson, Miss Annie Beaumont, Miss Jean Maxwell-Cunningham, Mrs. Gertrude Deakin, Miss Isabella Grassick, Miss Edith Hadfield, Miss Violet Jolly, Miss Margaret Lyons, Miss Florence Marsh, Miss Mary O'Brien, and Miss Edith Passmore, Q.A.I.M.N.S.(R.), Miss Ethel Atkin, Miss Charlotte Elgin, Miss Jane Fairgrieve, and Miss Catherine Macaulay, T.F.N.S.; Miss Margaret Robertson, B.R.C.S.; Miss Dorothy Dawson, Miss Jessie Duncan, Miss Charlotte Jones, Miss Florence Knobel, and Miss Neary Ronaldson, C.N.S. His Majesty conferred the Military Medal on Mrs. Agnes Parker, F.A.N.Y.; and Miss Vivien Mellor, V.A.D.

The following decorations and medals have been awarded by the Allied Powers to members of the British Nursing Services for distinguished service rendered in the course of the war. The King has given unrestricted permission in all cases to wear the decorations and medals in question: Conferred by the King of the Belgians.—*Médaille de la Reine Elisabeth*: Matron-in-Chief Dame E. M. McCarthy, G.B.E., R.R.C., Q.A.I.M.N.S. Conferred by the King of the Hellenes.—*Medal for Military Merit*: A large number of matrons and nursing sisters belonging to British and Australian Nursing Services. Conferred by the President of the Portuguese Republic.—*Order of Christ, Officers*: Sister G. A. Aitchison, Matron M. A. Harvey, R.R.C., Matron J. E. Hills, R.R.C. *Chevaliers*: Sister Barugh, A.R.R.C., Acting Sister V. I. Bryden, Sister M. A. Cracknell, A.R.R.C., Sister L. M. Duckett, Staff Nurse M. Elliott, Sister Alice Grandjean, Sisters M. J. Jessop, N. Hayes, A.R.R.C., E. Kerr, R.R.C., A. L. Molyneux, A.R.R.C., G. Morgan, Staff Nurse A. M. O'Shaughnessy, Staff Nurse E. A. Palmer, Sister H. E. Panton, M.M., Assistant-Matron V. Rogers, R.R.C., Staff Nurse E. Rothwell, Staff Nurse L. T. Wynn.

A very mischievous propaganda has been set on foot by an Association calling itself "The Mothers' Defence League," which is trying to stir up opposition to the work of school nurses, health visitors, almoners and other agents of the Health Ministry. They are attempting to do this under the name of liberty, and one cannot but remember "the crimes, O Liberty, which have been committed in thy name." Cannot these restless intriguers be brought to realise that a new future full of health and promise is opening out before the next generation? We older ones look back on a youth sadly maimed and saddened for lack of the opportunities now offered to the boys and girls among us. There is no other way in which the good message of hope and enlightenment can be brought to homes still darkened by age-long ignorance than by personal visiting undertaken by trained women whose purpose is free from self-interest. Doubtless the liberty of fathers and mothers is to some extent curtailed by the measures which set their children free to begin life healthy and well-instructed. It is a transition period, and the need is for patient spade-work of explanation and the removal of prejudices To foster prejudice and attempt to retard reform is work for which men and women should be ashamed to combine.

The ultimate aim of the many social organisations of the day is, or should be, to make the Englishmen's home "his castle," by the creation not only of a suitable structure, but of the right family atmosphere and home influence. So many factors contribute to this end, however, that the public is confronted with just so many different organisations, each aiming at doing their part. It has been realised for some time that the practical result of such a position is that one family may be the recipient of numerous visits from representatives of various social agencies. Social workers are all familiar with the story of the harassed mother with the half-dozen visitors in her house on the same day. It appears that protests are now being made by the people who have experienced such visitations, and that a deputation has been instructed in the legal rights of exclusion of certain visitors. Health visitors, school nurses, officers of the N.S.P.C.C., and presumably Care-Committee workers find their place among those who may be refused admission to the home. While feeling very strongly that the social work of a district should be done through one committee and one only, as on the "area" scheme, which has been successfully tried in one or more parts of London, we cannot but feel that it is a great pity to lay too much stress publicly on the matter. The more it is impressed upon people that they have this right of exclusion, the more likely they are to use it, and thus render the work of health-visitors, welfare-workers and the like more difficult than it already is. The tendency will be for an attitude of resentment to be fostered in place of the necessary one of mutual co-operation and understanding.

The meeting of the Hospital Linen League of Queen Mary's Hospital, Stratford, was a very cheerful function, at which Mrs. Lloyd George was present. There are over 1,000 subscribers, who have raised over £400, and have got together a splendid collection of gifts, mainly the work of their own hands. Mr. C. E. Leo Lyle, M.P., chairman of the hospital, expressed his thanks to the workers.

## Figures and Tables

**Figure f1:**