# Erratum

**Published:** 1920-01-10

**Authors:** 


### Erratum.

St. Thomas's Hospital.—On page 217 of The Hospital of December 6 it is stated that no nurse in this hospital is off duty for less than three and a-half hours daily. It should be less than three hours daily, which is correct.

